# The Yin and Yang of Discoidin Domain Receptors (DDRs): Implications in Tumor Growth and Metastasis Development

**DOI:** 10.3390/cancers13071725

**Published:** 2021-04-06

**Authors:** Sandra Majo, Patrick Auguste

**Affiliations:** Inserm Bmgic U1035, University of Bordeaux, F-33000 Bordeaux, France; sandra.majo@u-bordeaux.fr

**Keywords:** discoidin domain receptors, extracellular matrix, tumor growth, metastasis

## Abstract

**Simple Summary:**

The tumor microenvironment plays an important role in tumor development and metastasis. Collagens are major components of the extracellular matrix and can influence tumor development and metastasis by activating discoidin domain receptors (DDRs). This work shows the different roles of DDRs in various cancers and highlights the complexity of anti-DDR therapies in cancer treatment.

**Abstract:**

The tumor microenvironment is a complex structure composed of the extracellular matrix (ECM) and nontumoral cells (notably cancer-associated fibroblasts (CAFs) and immune cells). Collagens are the main components of the ECM and they are extensively remodeled during tumor progression. Some collagens are ligands for the discoidin domain receptor tyrosine kinases, DDR1 and DDR2. DDRs are involved in different stages of tumor development and metastasis formation. In this review, we present the different roles of DDRs in these processes and discuss controversial findings. We conclude by describing emerging DDR inhibitory strategies, which could be used as new alternatives for the treatment of patients.

## 1. Introduction

**Figure 1:**
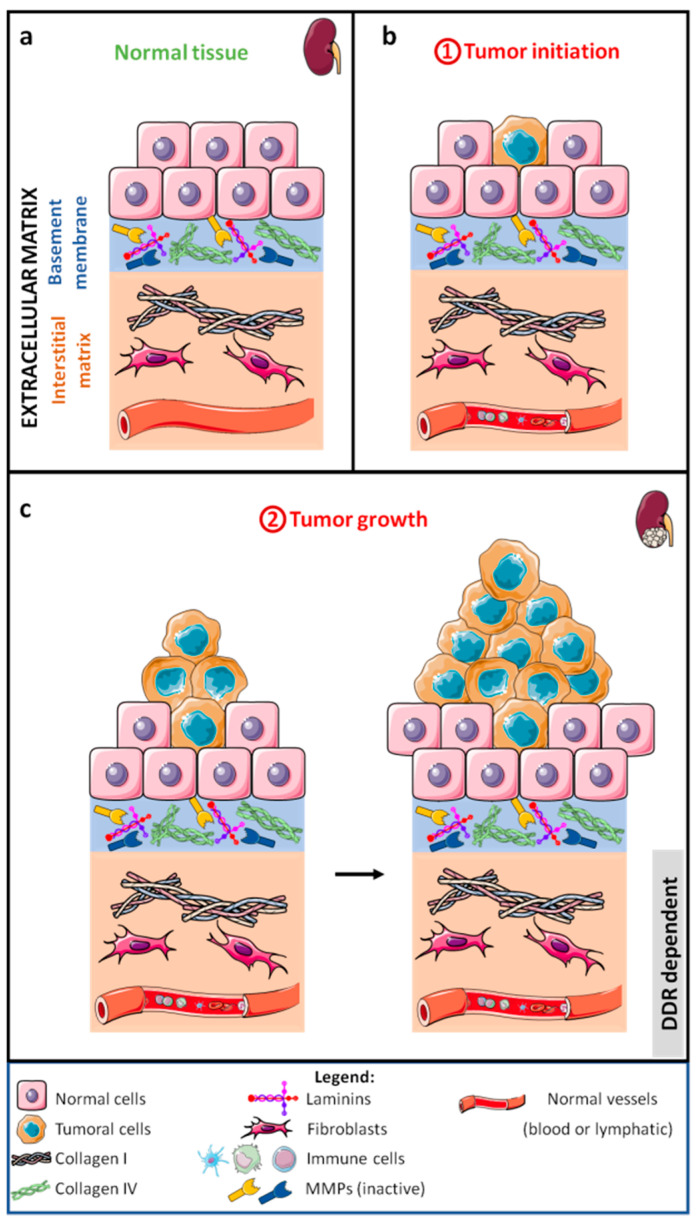
Schematic representation of normal tissue (**a**), carcinoma initiation (**b**), and initial stages of carcinoma development (**c**).

The development of tumors, particularly those of epithelial origin that lead to carcinoma (Figure 1a,b), begins with rapid and anarchic proliferation of cells, giving a tumor mass (Figure 1c). Soon, the tumor stops growing due to nutrient and oxygen deficiency.

**Figure 2:**
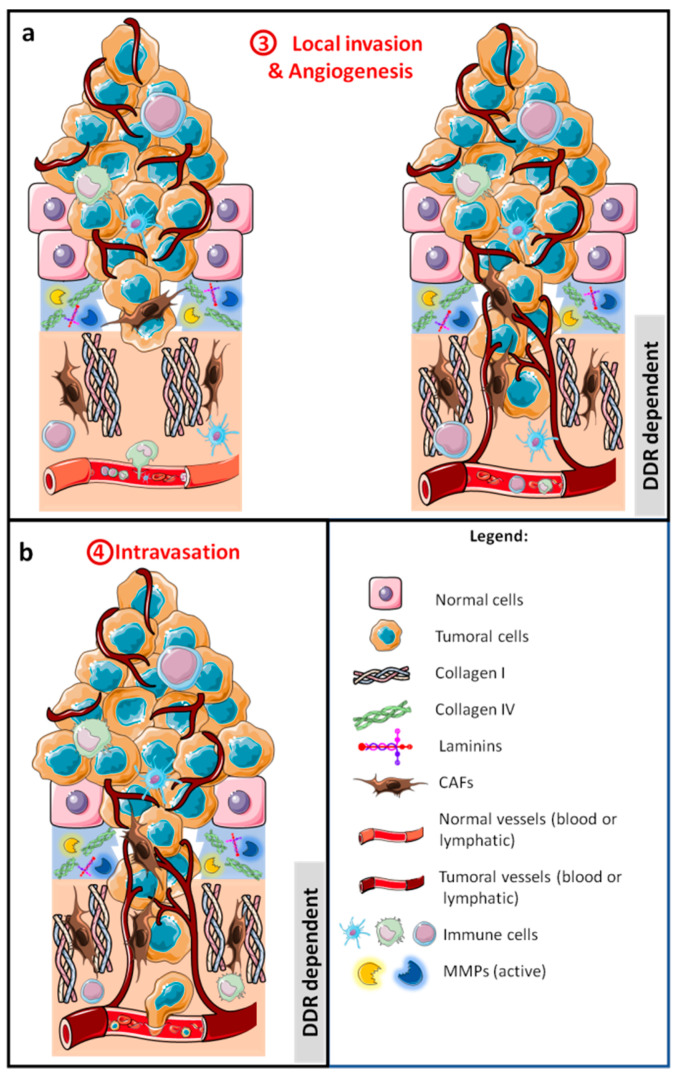
Schematic representation of the development of a carcinoma with the first metastatic stages, local invasion of tumor cells, and angiogenesis (**a**), allowing tumor cells to intravasate into lymphatic and/or blood vessels (**b**).

To grow further, the tumor cell mass attracts cells of different types such as endothelial, immune, and fibroblastic cells, together forming the tumor stroma or tumor microenvironment (TME, Figure 2a) [1]. At this stage, the primary tumor is well confined and surgical resection combined with adjuvant therapies can cure a vast majority of cancers. Nevertheless, tumor cells can escape from the primary tumor and colonize other organs in a process called metastasis.

**Figure 3:**
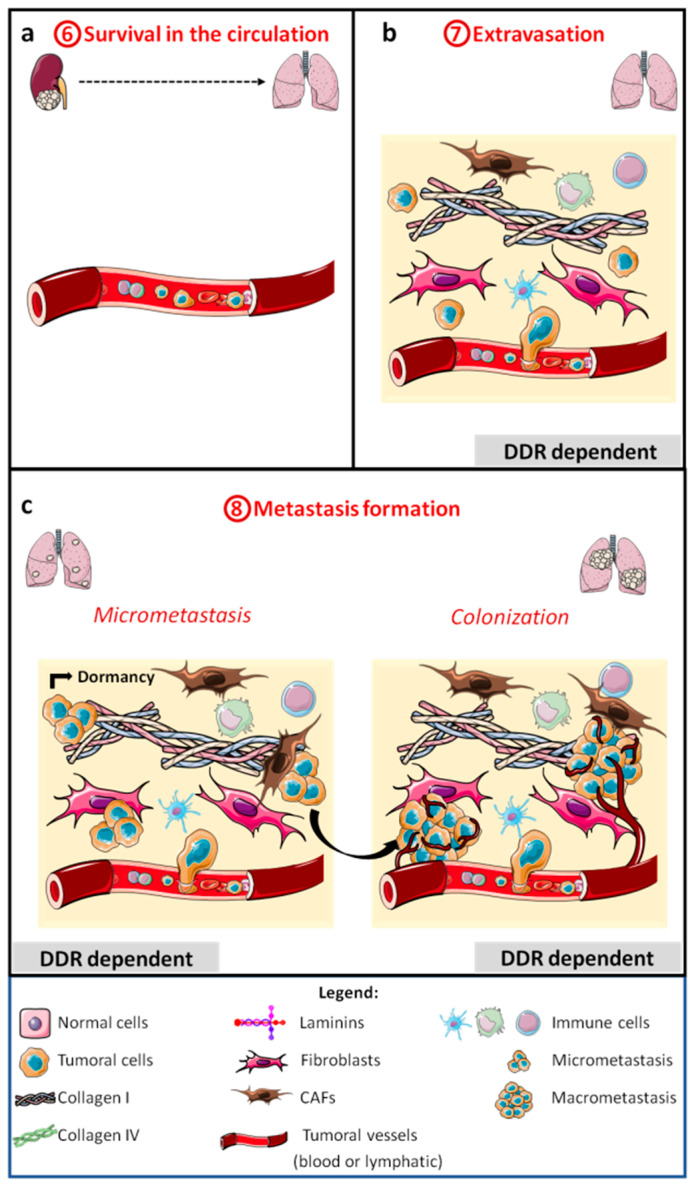
Schematic representation of the late metastatic stages: survival in the circulation (**a**), extravasation (**b**), micrometastasis, and colonization or macrometastasis formation (**c**).

The multistep metastatic cascade was established in 2003 by Fidler [2] and refined several times since then [3,4]. The first step in the cascade is the invasion by tumor cells of the extracellular matrix (ECM) and the multilayer of stromal cells, forming the primary tumor (Figure 2a). Then, to disseminate, tumor cells intravasate into lymphatics or blood vessels (Figure 2b) where they survive anoikis, flow pressure, and the presence of immune cells (Figure 3a).

Subsequently, tumor cells arrest in the vasculature of distant organs and extravasate (Figure 3b). In the target organ tissues, tumor cells survive in a hostile microenvironment and form quiescent micrometastasis (tumor dormancy) (Figure 3c), before growing again in a step called macrometastasis or metastatic colonization (Figure 3c). During this process, tumor cells are exposed to different microenvironments, including different ECMs.

The ECM, one of the noncellular components of the TME, is composed of proteins, glycoproteins, proteoglycans, and polysaccharides, and it can be divided into the basement membrane and interstitial matrix. Epithelial cells are surrounded by the basement membrane, which is mainly composed of laminins, type IV collagen, nidogen, and heparan sulfate proteoglycans. The basement membrane separates the epithelial cells from the interstitial matrix. The latter is composed of collagens, elastin, proteoglycans, fibronectin, and other proteins. As a whole, the ECM forms an interconnected network. During tumor development, the ECM is continuously remodeled, with new synthesis and proteolytic degradation of the matrix components [5]. Collagens are the most abundant proteins in the ECM and, to date, 28 different collagens (I to XXVIII) have been found expressed in tissues and tumors. All collagens have in common the presence of three α chains forming a triple-helicoidal domain. Depending of the collagen, the triple-helix domain represents a mass ranging from more than 90% (collagen I) to less than 10% (collagen XII) of the protein. The diversity of the collagens is increased by the existence of molecules with more than one chain in some of them [6]. Collagens are classified into six different families, such as fibril-forming collagens (I–III, V, XI, XXIV, and XXVII) and network-forming collagens (IV, VIII, and X) [6].

In most cancers, e.g., colorectal or breast cancer, high collagen expression is associated with a poor prognosis. However, in some cancers, collagens may play an important role in limiting tumor cell activation or in constituting an obstacle to tumor growth [7]. In addition, proteolytic fragments of some collagens, such as endostatin, may inhibit tumor growth [8]. These antagonistic roles need to be further investigated in the perspective of anti-collagen therapies [7]. Collagens are primarily responsible for tumor rigidity and can affect tumor behavior through mechanical processes, e.g., by providing either a barrier for tumor development or a track for tumor cell migration [9,10]. On the other hand, collagens can influence tumor cell behavior by binding to extracellular receptors that induce intracellular signaling. The major collagen receptors present in carcinoma cells are integrins, α1β1, α2β1, α10β1, and α11β1. Intracellular integrin signaling is primarily mediated by activation of FAK (Focal Adhesion Kinase) and SFK (Src Family Kinase) proteins. In addition to these well-known receptors, collagens can bind cluster of differentiation 44 (CD44) and discoidin domain receptors (DDRs) [11].

**Figure 4:**
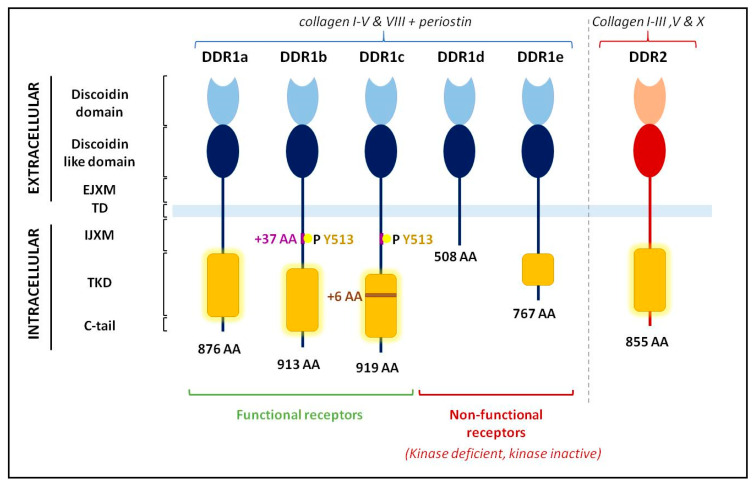
Schematic representation of the five discoidin domain receptor 1 (DDR1) isoforms and of the only DDR2 form. EJXM: extracellular juxta-membrane domain, TD: transmembrane domain, IJXM: intracellular juxta-membrane domain, TKD: tyrosine kinase domain, C-tail: C-terminal part of the receptors. Adapted from [12,17].

DDRs are a family of tyrosine kinase receptors (TKRs) with two members, DDR1 and DDR2. DDRs share an extracellular domain similar to the discoidin I lectin of the amoeba *Dictyostelium discoideum*, a discoidin-like domain, and an extracellular juxta-membrane domain (EJXM). The transmembrane domain is followed by an intracellular juxta-membrane domain (IJXM) and by the tyrosine kinase domain (TKD). DDR1 and DDR2 are encoded by single genes present in chromosome 6 (6p21.33) and 1 (1q23.3), respectively. Alternative splicing results in DDR1 isoforms, a, b, c, d, and e (Figure 4). DDR1b and c share a 37 amino-acid sequence inserted within the IJXM domain, compared to DDR1a. This stretch of 37 amino-acid insertion includes a tyrosine residue (Tyr 513) essential for DDR1 signaling. DDR1c has six extra amino acids in the kinase domain compared to DDR1b. DDR1a and b are the major isoforms expressed. Isoforms d and e are deleted or mutated in the TKD, resulting in inactive tyrosine kinase isoforms. In contrast, only one form of DDR2 is found (Figure 4) [12].

Both receptors bind to fibrillar collagens with certain ligand specificities; DDR1 preferentially binds to collagen I–V and VIII while DDR2 binds to collagen I–III, V, and X [13]. In addition, DDR1 is able to bind periostin, another component of the ECM [14]. In the absence of any ligand and as for other TKRs, a mixed population of DDR monomers and homodimers is expressed at the cell surface. However, unlike other TKRs, fibrillar ligand-induced DDR activation is very slow, taking hours to set up and persisting for more than 24 h. In this step, the DDRs cluster, the tyrosine kinase activity is induced, and the dimers phosphorylate each other [15,16].

Several tyrosine residues are phosphorylated, notably Tyr513 and Tyr792, 796, and 797 within a clustering DDR1b, allowing the recruitment of various adaptor proteins involved in signal transduction [17]. Recently, a noncanonical activation mode was proposed for DDR1b, in which the binding of monomeric collagen to DDR1b induces a rapid (30 min) clustering, leading to internalization and re-expression at the cell surface as Tyr513-phosphorylated DDR1b. Then, if fibrillar collagens are present, linear clusters of DDR1b are formed, and canonical activation of the receptors occurs with the phosphorylation of Tyr740 and 792. Since Tyr513 is not present in DDR1a and has no equivalent in DDR2, this noncanonical pathway is only possible with DDR1b and c [18,19]. Activation of DDR2 appears to be more complex and requires phosphorylation by Src kinase. Binding of collagen to DDR2 induces phosphorylation of Tyr740 by Src, thereby releasing the autoinhibitory activity of the kinase, allowing phosphorylation of other tyrosine residues and recruitment of adaptor proteins [20]. Heterodimers or at least heteroclusters between DDR1 and DDR2 have been found in coimmunoprecipitation assays [21]. DDRs activate various intracellular signaling pathways such as extracellular signal-related kinase (ERK), signal transducer and activator of transcription (STAT), Src, protein kinase B (AKT), or Yap [17,21,22,23].

Nevertheless, DDRs can induce intracellular signaling independent of tyrosine kinase activity or of collagen binding. The formation of linear invadosomes is mediated by collagen binding to DDR1 and activation of the Cdc42-GEF (guanine nucleotide-exchange factor) Tuba, and it is independent of tyrosine kinase activity [24]. The involvement of DDR1 in the process of collective invasion is collagen-independent and only requires DDR1 interaction with E-cadherin to induce activation of Par3 and 6 [25]. DDR1 is also able to interact with other partners such as the tetraspanin-like protein TM4SF1 (Transmembrane 4 L Six Family Member 1) to mediate intracellular signaling [26]. Less is known about DDR2 interactions with its partners.

The regulation of DDR expression is not well understood. Hypoxia is a positive regulator of DDR1 and DDR2 expression in nontumor cells, such as mesenchymal stem cells and smooth muscle cells [27,28], and in pituitary adenoma and breast tumor cells [29,30]. The transcription factors Zeb1 and Twist1, involved in the epithelial-to-mesenchymal transition (EMT) process, increase the expression of DDR1 or DDR2, respectively [31,32]. E2F1 and P53, two cell-cycle transcription factors, increase DDR1 messenger RNA (mRNA) expression in different tumor cell lines [33,34]. In hepatocellular carcinoma, DDR1 activates STAT3 which, in turn, increases DDR1 mRNA expression [35]. ATF4 (Activating Transcription Factor 4) mediates osteoclast differentiation by increasing DDR2 gene transcription [36]. Angiotensin II and TNFα (Tumor Necrosis Factor alpha) increase DDR2 mRNA expression [37,38], whereas TGFβ1 increases DDR1 at both mRNA and protein level [39]. In lung fibroblasts, collagen I activates DDR2 and, in turn, JAK2 (Janus Kinase 2) and ERK1/2 signaling pathways leading to up-regulation of DDR1 [40]. MicroRNAs (miRNAs) are potent regulators of DDR expression, with miR-199-a/b 5p [41] and miR-486-3p [42] for DDR1 and miR-615-5p [43] for DDR2. Lastly, a growing body of evidence suggests epigenetic control of DDR1 mRNA expression. In patients with idiopathic nonobstructive azoospermia or ovarian or lung cancers, DDR1 expression was found to correlate with the methylation of the promoter [44,45,46].

DDR1 activity may be regulated by a shedding mechanism. Collagens are able to activate DDR1 and some batimastat-sensitive proteinases. In turn, active proteinases, such as MT1-MMP-1, MT2-MMP, MT3-MMP (Membrane Type Matrix Metalloproteinases), and a disintegrin and metalloproteinase (ADAM) 10 cleave DDR1 at EJXM, leaving a 62 kDa intracellular membrane anchored protein. This cleavage is responsible for inhibiting the tyrosine kinase activity of DDR1, but nothing is known about the function of the extracellular part of DDR1 [13,47,48].

In this review, we focus primarily on the involvement of DDRs in carcinoma development and metastasis. We discuss the potential roles of these receptors in certain stages of metastasis.

## 2. Roles of DDRs in Cancer Progression

### 2.1. Tumor Growth

#### 2.1.1. DDRs and Tumor Cells

The transformation of a normal cell into a cancer cell involves different mechanisms such as gene activation/inactivation and chromosomal abnormalities. DDR1 is not important for tumor initiation, but it is critical in early tumor development and progression in KRAS (Ki-ras2 Kirsten rat sarcoma viral oncogene homolog)-driven lung adenocarcinoma [49] (Figure 1a,b). DDR1 is not expressed in normal gastric epithelial cells, but it is expressed in half of gastric carcinoma, showing that DDR1 is an important mediator of gastric cancer aggressiveness [50]. DDR2 has been shown to be a cancer-related gene associated with prostate cancer aggressiveness and progression, and its expression is upregulated in advanced benign prostate hyperplasia and prostate cancer compared to normal tissue [51]. This involvement of DDRs in the development and aggressiveness of tumors translates into an effect in several biological processes. Thus, numerous studies have shown that DDRs have an impact in vitro and in vivo on tumor growth by promoting or inhibiting proliferation and apoptosis in a cancer- and context-dependent manner, alone or in combination with other molecules (Figure 1c).

DDR1 has been shown to play a critical role in promoting the proliferation of several cancer cells, in vitro and in vivo. In different cancer cell lines, DDR1 promotes survival by regulating the Ras/Raf/MEK/MAPK (Rat sarcoma/Rapidly Accelerated Fibrosarcoma/Mitogen-activated protein kinase kinase/Mitogen-activated protein kinase) pathway, resulting in positive feedback on the expression of the tumor suppressor P53 and the activation of AKT [34]. In Hodgkin’s lymphoma, latent membrane protein 1 (LMP1) induces DDR1 expression in germinal center B cells and protects tumor cells from death [52]. In prostate cancer, prostate cancer antigen 1 (PCA-1) increases the level of DDR1 and Bcl-xl, an antiapoptotic molecule, resulting in the inhibition of apoptosis [53]. In non-small-cell lung carcinoma (NSCLC), DDR1 induces tumor cell proliferation in vivo. Inhibition of DDR1 or TMPRSS4 (Transmembrane protease serine 4), a membrane-bound serine protease, reduces proliferation to various extents with little alteration of cell-cycle progression or apoptosis. These effects are potentiated when DDR1 and TMPRSS4 are co-inhibited, resulting in complete inhibition of proliferation with disappearance of cyclin A and B1, increased P21 expression, and gap 0 (G0)/G1 cell-cycle arrest [54]. DDR1 can also promote proliferation in vivo. For example, inhibition of DDR1 in a subcutaneous gastric cancer xenograft slows tumor growth [50]. Furthermore, in pancreatic ductal adenocarcinoma, tumors from DDR1^−/−^ mice have significantly fewer Ki-67^+^ proliferative cells [55]. This is consistent with an in vitro study of pancreatic cancer cells which showed that DDR1 inhibits TGFβ1 expression, thereby promoting tumor cell proliferation [56]. The role of DDR1 in breast cancer proliferation or survival appears to depend, at least in part, on tumor type or culture method. In vitro, targeting DDR1 expression inhibits proliferation of T47D and MCF-7 luminal cells or MDA-MB-435 triple-negative cells [57,58]. In contrast, in other breast cancer studies, DDR1 was found to be a proapoptotic receptor. Luminal breast cancer MCF-7 cells and basal-like breast cancer MDA-MD-231 cells express high and low levels of DDR1 and low and high levels of MT1-MMP, respectively. When cells are cultured in a three-dimensional (3D) collagen matrix, DDR1 in MCF-7 cells is involved in apoptosis and cell growth inhibition through upregulation of the proapoptotic mediator BIK (Bcl-2-interacting killer) [59]. Conversely, in MDA-MD-231 cells, MT1-MMP has a protective effect through degradation of collagen I and cleavage of DDR1, altering the collagen/DDR1/BIK pathway to induce apoptosis and suppress tumor growth [60]. In colon cancer, DDR1 interacts with LRP1, a lipoprotein receptor inducing DDR1 endocytosis, thereby controlling its expression at the plasma membrane. The DDR1/LRP1 interaction increases 3D cell proliferation and cell-cycle progression, as well as decreases apoptosis [61].

DDR2 has been shown to promote proliferation in human melanoma [62], oral squamous cell carcinoma [63], gastric cancer [64], hepatocellular carcinoma [65], lymphoma [43], and lung cancer [66] among others. Indeed, in vitro inhibition of DDR2 expression results in decreased proliferation in melanoma cell lines via JNK (c-Jun N-terminal kinase) phosphorylation [62] and induction of apoptosis in hepatocellular carcinoma cells [65]. In oral squamous cell carcinoma, downregulation of colon cancer-associated transcript 1 (CCAT1) prevents proliferation via inhibition of DDR2, resulting in inactivation of ERK/AKT pathways [63]. Lastly, Circ-LAMP1 (Lysosomal-associated membrane protein 1), the circular RNAs most expressed in T-cell lymphoblastic lymphoma, is capable of promoting proliferation by inhibiting apoptosis through modulation of miR-615-5P and its target DDR2 [43]. DDR2 has also been shown to enhance tumor growth in vivo. Indeed, in hepatocellular carcinoma xenograft, small interfering RNA (siRNA)-mediated inhibition of DDR2 expression in SNU182 cells led to decrease tumor growth [65]. In gastric cancer, overexpression of DDR2 promotes tumorigenesis in vivo by inducing tumor growth [67]. On the contrary, DDR2 may also inhibit cell proliferation in melanoma and fibrosarcoma cells in vitro. In these cells, fibrillar collagen activates DDR2 resulting in inhibition of proliferation characterized by growth arrest in the G0/G1 phase of the cell cycle [68].

Tumor cell proliferation can be modulated by DDR mutations. Indeed, some mutations have been identified in DDR1 and DDR2 throughout their structures, but the impact of mutations has been studied mainly for DDR2 and more particularly in lung cancer. DDR2 is mutated in 3–4% of cases. Some mutations such as E655K play a role in tumor progression by weakening the growth-inhibitory effect induced by collagen via DDR2, while V582E or L595F mutations also increase colony size in vitro [69,70].

As shown above, the impact of DDRs may be different in in vitro and in vivo situations, and it may depend on the structural organization of the TME. In the case of breast carcinoma [71], in vitro inhibition of DDR1 promotes tumor growth only in 3D. The rigidity of the microenvironment plays a critical role and can either activate or inhibit proliferation and invasion. The NSCLC cell line H1299 grown on a soft matrix expresses p300 at low levels, leading to reduced acetylation of c-myb and weak interaction between the DDR2 promoter and c-myb or LEF1 (Lymphoid enhancer-binding factor-1). Under these conditions, the DDR2 promoter is not very active, resulting in low proliferation and invasion. On the contrary, when the matrix stiffens, the DDR2 promoter is highly active, resulting in increased proliferation and invasion [66]. Table 1 summarizes the different roles of DDRs in cancer-cell survival or proliferation.

The TME is not only composed of cancer cells but also of stromal cells such as CAFs. An interaction between DDRs and CAFs has been established in several cancers. In gastric carcinoma, CAFs induced the upregulation of DDR1 in tumor cells in vitro by potently activating STAT3, increasing its tumorigenic potential. In vivo, on gastric carcinoma, pharmacological inhibition of DDR1 in the mouse xenograft model impeded CAF-induced tumorigenesis with a reduction in the number of tumor nodules [72].

**Table 1:** Roles of DDRs in tumor cell survival or proliferation. NSCLC, non-small-cell lung cancer; SCC, squamous cell carcinoma.

Receptor	Cell Survival or Cell Proliferation	Cancers
DDR1	increase	-Sarcoma [34] -Colon carcinoma [34] -Breast carcinoma [57,58] -Hodgkin’s lymphoma [52] -Prostate carcinoma [53] -Lung (NSCLC) [54], (adenocarcinoma) [49] -Gastric carcinoma [50] -Pancreatic carcinoma [55,56]
	decrease	-Breast carcinoma [59,60,71] -Colon carcinoma [61]
DDR2	increase	-Melanoma [62] -Oral SCC [63] -Gastric carcinoma [64,67] -Hepatocellular carcinoma [65] -Lymphoma [43] -Lung (NSCLC) [66]
	decrease	-Melanoma [68] -Fibrosarcoma [68,73]

#### 2.1.2. DDRs and Immune Cells

The immune system plays an essential role in the response against the tumor. In order to stop tumor progression, this response is mediated by T and B lymphocytes, natural killer cells, macrophages, and dendritic cells. The immune cells migrate through the matrix where collagen is very abundant and enter the bloodstream to reach the tumor site. In 2011, two new characteristics of cancer were revealed and, among them, is the escape from the immune system [74]. DDRs have been shown to control certain characteristics of immune cells.

DDR1 is able to enhance the migration of the T helper 17 (Th17) population of T lymphocytes in 3D collagen matrices through different pathways: RhoA/ROCK/MAPK/ERK (Ras homolog family member A/ Rho-associated protein kinase/ Mitogen-activated protein kinase/ Extracellular Signal-Regulated Kinase) in vitro and in vivo [75] or activation of p38 MAPK [76]. Recently, in breast cancer, an inverse correlation was made between DDR1 expression by tumor cells and the level of CD4^+^ and CD8^+^ T-lymphocyte infiltration. The molecular mechanism involved in these situations is not known [77].

DDR2 plays an important role in the collagen-mediated differentiation of dendritic cells to a mature phenotype producing a high amount of IL-12 (Interleukin-12). DDR2 enhances the functional abilities of these mature dendritic cells to activate T cells, thereby regulating immune responses [78]. While integrins are important mediators of neutrophil migration on a 2D collagen matrix, DDR2, but not DDR1, is involved in neutrophil migration in 3D collagen. DDR2 regulates neutrophil directionality through increased secretion of MMP8 [79]. Furthermore, in adipose tissue, T-cell activity is controlled by myeloid-derived CD45^+^/DDR2^+^ cells. These CD45^+^/DDR2^+^ cells express increased levels of MHC II (major histocompatibility complex II) and CD80, two markers of activation and antigen presentation, and lead to increased production of the proinflammatory cytokines, IFN-γ (Interferon gamma) and TNF-α, by CD4^+^ T cells [80]. The presence and the role of these myeloid-derived CD45^+^/DDR2^+^ cells in tumor progression are not known.

### 2.2. Local Invasion

#### 2.2.1. Extracellular Matrix Breakdown

In order to spread into different tissues, tumor cells must first breach the surrounding basement membrane, which is mainly composed of collagen IV and laminins. The capacity of tumor cells to overcome the basement membrane is decisive for tumor progression (Figure 2a) [81]. DDRs are able to modulate the expression of MMPs, in particular MMP2 and MMP9, which are involved in the remodeling and degradation of the basement membrane.

In lung cancer, DDR1 inhibition decreases the activity of MMP2 and MMP9 [82]. Modulation and regulation of MMP2 and/or MMP9 activity by DDR1 have also been demonstrated in pituitary adenoma [83], colorectal cancer [84], and renal cancer (MMP2) [85]. In pancreatic cancer, a novel pathway including TM4SF1/DDR1/MMP2 and 9 enhances invadosome formation and activity, thereby enhancing cell migration and invasion capabilities [86]. In liver cancer, the interaction between C1q, a component of the complement system, and DDR1 induces the upregulation of MMP2 and MMP9 expression, leading to migration and invasion [87]. C1q is present in the microenvironment of many tumors such as colon, pancreatic, breast, lung adenocarcinoma, and melanoma, and it is expressed by stromal cells such as fibroblasts [88].

Like DDR1, DDR2 is involved in the modulation of MMP expression in ovarian cancer where it regulates the expression of MMP1–3, 7, and 13 mRNAs and the activity of MMP2 and MT1-MMP [32]. In human melanoma cells, downregulation of DDR2 is associated with decreased MMP2 and MMP9 activities [62]. Overexpression of DDR2, through ERK2/Snail signaling, increases MT1-MMP and MMP2 expression in hepatocellular carcinoma [89] and MT1-MMP expression in breast cancer [90]. Nevertheless, DDR2 was found to be required for collagen I-mediated activation of MT1-MMP in fibroblast but not in fibrosarcoma or in breast cancer [91].

Basement membrane degradation is mediated by the formation of specialized structures called invadosomes which degrade the basement membrane while maintaining cell-cell contacts and tissue integrity [92]. The involvement of DDRs in the formation of these structures has been demonstrated in several studies. In breast cancer cells, the binding of collagen I to DDR1 leads to the activation of the Cdc42-GEF Tuba, thereby inducing the formation of linear invadosomes, as well as promoting the activation of the proteolytic machinery and cell invasion. This process is independent of the tyrosine kinase activity of DDR1 [24]. In hepatocellular carcinoma, DDR1-induced linear invadosome formation is enhanced in the presence of TGFβ1 [39].

DDR2 is required to elaborate linear invadosomes in endothelial cells and to facilitate matrix degradation in association with VEGF (Vascular Endothelial Growth Factor), but it is not known if DDR2 plays the same role in cancer cells [93]. Table 2 summarizes the different roles of DDRs in MMP activation and invadosome formation.

After basement membrane degradation, cells encounter collagen I in the interstitial matrix (Figure 2a). Aligned collagen oriented perpendicular to the tumor boundary has been shown to promote invasion, and it is considered as a poor prognostic marker for survival or recurrence in patients with breast carcinoma [94]. Three tumor-associated collagen signatures (TACS) have been defined. TACS-1 is characterized by increased collagen deposition near the tumor that appears very early in tumor formation. As the tumor grows, TACS-2 and -3 have been defined. TACS-2 corresponds to collagen fibers stretched around the tumor, and TACS-3 is the situation in which collagen fibers are aligned perpendicular to the tumor, promoting local invasion [95]. DDR1 and DDR2 participate in collagen fiber alignment and have been associated with TACS-3 [90,96,97,98]. Accordingly, inhibition of DDR2 expression decreases collagen deposition and fiber alignment and thereby tumor growth, migration and invasion are reduced [90,99].

The structure of collagen I can be impacted by aging, affecting many biological processes. DDR1 is very sensitive to these changes and, consequently, the regulation of proliferation and apoptosis is altered. For instance, the proteolytic degradation of collagen I by MT1-MMP, which increases with aging, was shown to decrease DDR1 activation, tumor growth, and inhibition of apoptosis [71]. This effect is also observed with DDR2; collagen I aging reduces DDR2 activation, resulting in induction of fibrosarcoma cell proliferation. In the presence of old collagen I, DDR2 and the tyrosine phosphatase SHP-2 (Src homology region 2-containing protein tyrosine phosphatase 2) are activated to a lesser extent, JAK2 and ERK1/2 remain phosphorylated, and expression of the cell-cycle negative regulator p21^CIP1^ decreases [73].

DDR2 expressed by cells of the TME is also involved in collagen remodeling.Inhibition of DDR2 in CAFs or in mesenchymal stem cells impacts their mechanotransduction function, disturbs collagen fiber organization, and decreases matrix stiffness and TACS-3 signature, leading to inhibition of breast tumor cell invasion [97,99].

#### 2.2.2. Epithelial–Mesenchymal Transition

EMT is a critical process involved in cancer development and progression. Depending on the context, this mechanism can lead epithelial cells to undergo multiple intermediate steps accompanied by physiological and morphological changes, transforming them in extreme cases into mesenchymal cells. EMT can be reversed by an event called mesenchymal-to-epithelial transition (MET). Both transitions are regulated by the induction or repression of transcription factors such as Zeb1/2, Snail1/2, and Twist among others, as well as the modulation of the expression of epithelial (E-cadherin) and mesenchymal (N-cadherin, vimentin, MMP9) markers [100]. DDRs have been shown to be associated with EMT in several cancers.

Some studies highlighted that DDR1 can induce EMT in vitro. For instance, in prostate cancer, DDR1 activates Pyk2 and MKK7 (Mitogen-activated protein kinase kinase 7), resulting in increased expression of the mesenchymal markers vimentin and N-cadherin and decreased expression of the epithelial marker E-cadherin [101]. In gastric cancer, a correlation among DDR1, E-cadherin, and vimentin has been demonstrated in cancer tissues. Overexpression of DDR1 increases the expression levels of vimentin and Snail1 and decreases that of E-cadherin [102]. Moreover, a negative correlation between miR-221-5p and DDR1 has been demonstrated. Overexpression of miR-221-5p in gastric cancer cells inhibits not only EMT, but also proliferation and invasion through modulation of DDR1 expression [103]. DDR1 induces EMT in hepatocellular carcinoma by regulating STAT3 in vitro and in vivo [35]. In colorectal cancer, downregulation of miR-199a-5p increases DDR1 expression, resulting in increased expression of mesenchymal markers [84]. In renal cancer OS-RC-2 and ACHN cell lines, collagen-mediated activation of DDR1 leads to increased expression of mesenchymal markers (vimentin/N-cadherin) and decreased expression of epithelial markers (E-cadherin) [85].

Conversely, several studies have shown that DDR1 expression is decreased during EMT. In breast cancer, a negative correlation between DDR1 and Zeb1 was found in carcinoma tissues. In the tumor, H-Ras is able to induce EMT by increasing Zeb1 and decreasing miR-200c expression. Zeb1 leads to suppression of E-cadherin expression and a reduction in DDR1 expression [31]. This negative correlation between Zeb1 and DDR1 is significantly associated in a female-specific manner in breast, ovarian, and liver cancers but not in lung or colon cancers [104]. Similarly, in epithelial ovarian cancer, a decrease in DDR1 expression is observed during the EMT process due to CpG hypermethylation of its promoter, and DDR1 inhibition did not affect E-cadherin expression at the protein level [45]. The effect of DDR1 on EMT inhibition has also been demonstrated in vivo in breast cancer. Crossing DDR1^−/−^ and MMTV-PyMT mice revealed that a loss of DDR1 expression increases the level of vimentin in the primary tumor while E-cadherin expression decreases. In the absence of DDR1, tumor cells exhibit a basal phenotype leading to enhanced invasion and are associated with the development of breast cancers of poor prognosis [105]. In the Madin-Darby Canine Kidney (MDCK) cell line, Slug (Snail2) induces EMT with no detectable level of E-cadherin and increases the level of mesenchymal markers (MMP9 or vimentin). During this transition, a switch from DDR1 to DDR2 expression is observed. Indeed, MDCK cells expressing Slug show a decrease in DDR1 expression accompanied by an increase in DDR2 expression [106]. This notion of switching between DDR1 and DDR2 has been recently suggested in ovarian cancer, where DDR2 mRNA expression is low in epithelial-like cells and increases in mesenchymal-like cells. Nevertheless, DDR2 protein expression could not be found in these mesenchymal-like cells [45].

The role of DDR2 in EMT activation is less controversial. An association between DDR2 and Snail1 has been revealed in several studies on breast cancer [30,90,98], ovarian cancer [32], papillary thyroid carcinoma [107], and hepatocellular carcinoma [89] via different signaling pathways. Indeed, immunohistochemistry on breast cancer tissues revealed an association of DDR2 expression with nuclear localization of Snail1, as well as with a loss of E-cadherin expression. The tyrosine kinase activity of DDR2 induces stabilization of Snail1 protein and involves stimulation of ERK2 in a Src-dependent manner. Importantly, in this study, DDR2 was induced during EMT but was not required for EMT induction [90]. Similarly, in hepatocellular carcinoma, DDR2 stabilizes Snail1 through ERK2 activity [89]. In gastric cancer, DDR2 promotes EMT by modulating the mTORC2 (mechanistic target of rapamycin complex 2)/AKT pathway [67].

Although a link has been established between DDRs and some factors modulating the EMT process, especially in vitro where a few signaling pathways have started to emerge, the mechanisms involved need to be further elucidated in vivo. Table 3 summarizes the different roles of DDRs in EMT.

#### 2.2.3. Migration and Invasion

DDRs have been extensively studied for their impact on migration and invasion. There are two main mechanisms of migration: cells moving individually (amoeboid and mesenchymal migration) or collectively in a cohesive multicellular unit (collective migration). Migration can be influenced by cytoskeletal organization, remodeling of the surrounding matrix by migrating cells, and cell–matrix interaction and force generation [108]. Adhesion to the ECM is important for cancer cells to migrate and, subsequently, to disseminate to different tissues. Some studies have shown that adhesion can occur in a DDR-independent manner, e.g., in breast cancer, the adhesion of MCF-7 cells to collagen is predominantly driven by β1 integrin rather than DDR1 [109]. Recently, using different combinations of HEK293T cell populations expressing either DDR1 and/or DDR2, we showed that the extracellular part of DDR1 or DDR2 is sufficient to mediate cell adhesion to collagen I in absence of integrin activities [21]. In human A375 melanoma, HT29 colon carcinoma and SK-HEP hepatoma cells, inhibition of DDR1 reduces by 75% very early tumor cell adhesion to collagen I with significant impairment of the cell–cell adhesion molecules ICAM1 (InterCellular Adhesion Molecule 1) and VCAM1 (vascular cell adhesion molecule 1) [110]. In prostate cancer, overexpression of DDR2 improved adhesion to collagen I [111].

DDRs are involved in migration and invasion processes as shown in numerous in vitro studies.DDR1 modulates the organization of the cytoskeleton and, consequently, has an impact on cell migration. In squamous cell carcinoma, DDR1 is localized at cell–cell contacts in association with E-cadherin and is able to regulate cytoskeletal organization, particularly actomyosin organization, and collective migration. The DDR1/E-cadherin/Par3/Par6 complex controls the localization of RhoE which antagonizes Rho/ROCK, modulating actomyosin contractility at cell–cell contacts. At these sites, actomyosin forces are weaker, allowing coordinated movement of the clustered cells [25]. In oral squamous cell carcinoma, DDR1 is involved in collective migration and invasion into a collagen matrix, leading to an angiolymphatic invasion in vivo [112]. In hepatocellular carcinoma cells, a cross-talk between DDR1 and STAT3 enhances tumor progression by promoting proliferation, migration and invasion in vitro, and tumorigenesis in vivo [35]. In pancreatic cancer, TM4SF1-induced migration and invasion require DDR1. Overexpression of DDR1 increases cell ability to form invadosomes to degrade the matrix and increases MMP2 and MMP9 [86]. In breast cancer cells, in a pathway involving the CD9 tetraspanin, DDR1 induces cell migration [113]. Collagen causes shedding of the DDR1 ectodomain by the metalloproteinase ADAM10. This ADAM10 and collagen binding-dependent DDR1 shedding is important for more efficient migration of epithelial cells on the collagen I matrix in vitro [48]. Consequently, migration is influenced by the control of DDR1 expression. In gastric cancer, miR-221-5p modulates DDR1 expression. When miR-221-5p is downregulated, DDR1 expression increases and promotes migration and invasion [103]. On the contrary, in pancreatic carcinoma, the interplay of DDR1 with E-cadherin and the Par3 signaling pathway was shown to prevent 3D invasion by enhancing cell–cell adhesion [114].

DDR2 has also been shown to promote migration and invasion in many cancers. Inhibition of DDR2 expression in gastric cancer [64] or hepatocellular carcinoma [65] reduces migration and invasion. Inhibition of DDR2 expression in melanoma suppresses migration and invasion through decreased activation of the ERK1/2 and NF-κB (Nuclear Factor Kappa B) pathways and, consequently, reduced expression of MMP2 and 9 [115]. In oral squamous cell carcinoma, downregulation of CCAT1 prevents proliferation, migration, and invasion through inhibition of DDR2 leading to inactivation of ERK/AKT pathway [63]. In head and neck squamous cell carcinoma, overexpression of DDR2 enhances migration and invasion [116]. In breast cancer, DDR2 can induce tumor cell migration and invasion when expressed by tumor cells and/or by CAF or multipotent stromal cells (MSCs) [90,117].

Although, in most cases, DDR1 and DDR2 have been shown to promote migration and invasion, few studies have shown their involvement in inhibiting both processes. DARPP-32 (Dopamine- and cAMP-regulated phosphoprotein, Mr 32 kDa) binds to the IJXM domain of DDR1 and decreases collagen-stimulated invasion of breast cancer cells [118].

Very few studies have analyzed the impact of DDRs on migration and invasion in vivo; instead, they focused on the establishment of metastasis as in gastric cancer [119]. PCA-1 increased prostate cancer cell invasion through DDR1 and MMP9 expression in a chick chorioallantoic membrane (CAM) assay [53]. In colorectal cancer, NSD2 (Nuclear Receptor Binding SET Domain Protein 2) circular RNA inhibits miR-199b-5p expression, which leads to an upregulation of DDR1 expression and promotes migration and invasion in vitro and in vivo [120]. DDR2 increases metastasis in a peritoneal xenograft model of ovarian cancer [32]. In a mouse model of spontaneous breast tumor development, inhibition of DDR2 expression by gene ablation largely reduces metastasis without affecting lung tumor growth [99,117].

Table 4 summarizes the different roles of DDRs in EMT.

### 2.3. Intravasation

There are two different routes for tumor dissemination: blood vessels or lymphatic vessels (Figure 2b). Angiogenesis is the primary vascularization process and is defined as the formation of new blood vessels from existing vasculature [1]. Likewise, lymphangiogenesis is the formation of new lymphatic vessels from pre-existent vessels [121]. During these processes, tumor blood and lymphatic vessels have the particularities of being disorganized and permeable to cancer cells, which facilitate their passage into the blood or lymphatic streams and, subsequently, the establishment of metastasis by their dispersion in different tissues. Tumor and endothelial DDRs are involved in angiogenesis.

DDR1, in renal cell carcinoma and in gastric cancer cells, induces the secretion of angiogenic factors involved in endothelial cell tube formation in vitro [85,119]. In gastric cancer cells, depletion of DDR1 suppresses the expression of key angiogenic factors such as VEGF-A, VEGF-C, and PDGF-B (Platelet-Derived Growth Factor B) [119] and leads to necrosis in vivo, as a consequence of impaired angiogenesis [102,119]. A direct role of DDR1 in angiogenesis was demonstrated by inhibiting DDR1 cell surface expression in microvascular endothelial cells (HMEC-1) [122].

DDR1 is also important for lymphangiogenesis. Gastric tumors develop fewer lymphatic vessels if DDR1 expression is inhibited [119] and lymphatic endothelial cells are unable to form tubes when DDR1 expression is suppressed by siRNA or with miR199a/b-5p [123].

In contrast to endothelial cells, in colon carcinoma, DDR2 is weakly expressed at the surface of normal endothelial cells; however, overexpression of the receptor enhances angiogenesis in vitro and in vivo. After injection of VEGF-containing Matrigel, angiogenesis is impacted in the *slie* mouse model carrying a deletion in the *DDR2* gene. When melanoma cells are injected, tumor development is impacted with decreased tumor angiogenesis and increased tumor necrosis; nevertheless, the remaining vasculature is mature and functional. In these tumors, proangiogenic factors or receptors such as VEGFR2, Ang-2 (Angiopoietin-2) or MMP9 are decreased, and antiangiogenic factors such as Ang-1 are increased [124]. In hepatocellular carcinoma, an association between DDR2 and VEGF has been found. Indeed, during hypoxia, DDR2 can regulate the VEGF pathway [125].

Nothing is known about the role of DDRs in the mechanism of intravasation.

### 2.4. Survival in the Circulation, Extravasation, and Micrometastasis Formation

No evidence for a role of DDRs in tumor cell survival in the bloodstream or extravasation has been established (Figure 3a–c). Nevertheless, in a model of liver metastasis, Yuge and collaborators injected gastric cancer cells into the spleen of mice and found no difference in the number of liver micrometastases whether or not cells expressed DDR1. These data suggest that there is no difference for tumor cell survival in the bloodstream or tumor cell extravasation to the liver parenchyma [119]. On the contrary, DDR1 in tumor cells is important for the migration of lung cancer cells to the bone niche after intracardial injection. However, it is not clear which stage of metastasis (survival in blood, extravasation, and/or micrometastasis) is under the dependence of DDR1 [82].

Recently, DDR1 expressed by the liver metastatic niche was found to be important for micrometastasis implantation. DDR1 siRNA-injected mice have fewer hepatic stellate cells (HSCs), differentiated myofibroblasts, and angiogenesis (CD31-positive liver sinusoidal endothelial cell (LSECs)). Consequently, less collagen is secreted into the niche and the number of micrometastases is reduced [126].

The importance of DDR2 in the metastatic niche depends of the metastatic tissue. In a model of liver metastasis, intrasplenic injection of colon cancer cells into DDR2^−/−^ mice showed an increase in micrometastasis foci compared to a DDR2^+/+^ mice. This result can be explained by increased HSC differentiation into myofibroblasts and increased LSEC activation and angiogenesis in DDR2^−/−^ mice. Furthermore, tumor cell adhesion to LSECs from DDR2^−/−^ mice is increased if these cells are exposed to tumor cell supernatants, suggesting that this may cause an increase in tumor cell extravasation in vivo. Lastly, the liver tumor niche in DDR2^−/−^ mice promotes tumor cell colonization (see below) [127]. In contrast, in a model of lung metastasis, intravenous tail injection of melanoma cells in *slie* mice showed a reduction in the number of lung metastases [124].

However, different types of collagen are found at higher levels in the serum from patients with cancer, and serum collagen IV is a biomarker for peritoneal dissemination of gastric cancer [128]. In breast cancer, HSP47 induces collagen secretion in the bloodstream where it can bind to circulating tumor cells and platelets, thereby promoting metastases [129]. C1q, which is part of the complement activation complex, contains a collagen-like domain. It was recently shown that DDR1 is activated in the presence of C1q, with phosphorylation at Tyr513 of DDR1 in hepatocellular carcinoma [87]. Furthermore, DDRs are involved in the activation of the pro-survival AKT pathway [130,131]. It is tempting to speculate that serum collagens and C1q can activate DDRs in circulating tumor cells to induce their survival in the blood stream.

The involvement of DDRs in tumor cell extravasation is still poorly documented.

### 2.5. Macrometastasis (Colonization)

The first demonstration of DDR1 involvement in tumor colonization (Figure 3c) was elegantly established by the Giancotti group [26]. In lung micrometastases, breast tumor cells produce and deposit collagen I in the ECM. Collagen can bind to DDR1 inducing the recruitment of the tetraspanin TM4SF1. In turn, TM4SF1 induces a large clustering of DDR1 and brings PKCα bound to the adaptor protein syntenin-2 in close proximity to the intracellular part of DDR1. PKCα phosphorylates and activates JAK2 and, consequently, STAT3 is phosphorylated and can, in turn, activate the transcription of genes such as *Sox2*. This mechanism of DDR1-induced metastatic colonization is independent of its tyrosine kinase activity. The formation of large DDR1 clusters is crucial for this mechanism, because collagen IV, which induces only small-size clusters, is not involved in the colonization process. Inhibition of TM4SF1 alters not only the metastases of breast tumors to the lungs, but also to the brain and bone. However, the involvement of DDR1 in brain and bone metastasis has not been assessed [26]. Recently, it was shown that TM4SF1 can regulate AKT and ERK activation via DDR1 in lung carcinoma [130], but it is unclear whether or not these regulations require DDR1 tyrosine kinase activity and whether these pathways are involved in DDR1-induced colonization. DDR1 is involved in gastric liver metastasis colonization, and intrasplenic injection of DDR1-inactivated gastric tumor cells shows no difference in micrometastasis formation but an inhibition of metastasis colonization [119]. In contrast, DDR1b expression in fibrosarcoma cells completely inhibits the formation of lung macrometastases after intravenous tail injection of these cells, but the step in which the metastasis process is altered remains unknown [23].

DDR2 has also been found to play a role in metastasis formation; however, again, it is not clear which step(s) of the metastatic process is (are) involved. In addition, DDR2 expression in tumor cells and in the metastatic niche may influence tumor metastatic colonization. In lung metastases of breast tumor cells, DDR2 expression by tumor cells is important for metastasis development, but its expression in the metastatic niche is dispensable [117]. In contrast, melanoma cells require DDR2 expression in the lung metastatic niche and in tumor cells to develop metastatic colonization [62]. Intrasplenic colon carcinoma cells injected into DDR2^−/−^ mice develop a liver niche favorable for micrometastasis development, as well as for tumor colonization. DDR2^−/−^ HSCs exposed to colon cancer cell supernatants overexpress genes (IL-10, TGFβ, and VEGF-A) involved in immune suppression, angiogenesis, and cancer cell growth. Therefore, the number of proliferating tumor cells is increased in liver metastases if DDR2 is not expressed in the liver [127].

### 2.6. DDR Association and Crosstalk with Other Membrane Receptors

**Figure 5:**
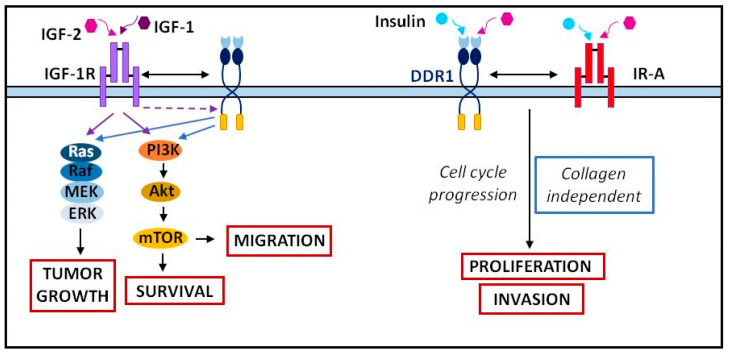
Crosstalk between DDR1 and the IGFs/insulin receptors. Adapted from [132,138].

The association or crosstalk of DDR1 with other transmembrane proteins results in distinct outcomes. Through a collagen- and tyrosine kinase-independent mechanism, DDR1 binding to E-cadherin increases the strength of cell–cell interactions. In breast cancer, some studies showed crosstalk between DDR1 and the insulin and IGF receptors, IR-A and IGF-1R (Insulin like Growth Factor 1 Receptor) (Figure 5). IGF-1 and IGF-2 stimulation lead to the upregulation of DDR1 by activating the PI3K (Phosphoinositide 3-kinase)/AKT pathway, thereby inhibiting miR-199a-5p expression. In the absence of collagen and in the presence of IGFs, DDR1 enhances the IR-A and IGF-1R pathways, thereby increasing tumor cell migration and survival through the PI3K/AKT pathway and tumor growth through the Ras/Raf/MEK/ERK pathway [132]. In addition, insulin or IGF-1 and IGF-2 induce an association between IGF-1R or IR and DDR1. DDR1 can also modulate IR expression by stabilization of both the mRNA and the protein [133]. In thyroid cancer, crosstalk between DDR1 and IGF-2/IR-A is responsible for tumor cell proliferation and invasion in 2D and 3D assays, and it is involved in the maintenance of tumor cell stemness [134]. In bladder cancer, migration and anchorage-independent growth are dependent on DDR1/IGF-1R or DDR1/IR-A crosstalk [135]. In A549 lung cancer cells, as collagen I does, the binding of IGF-1 to its receptor IGF-1R mediates DDR1-induced cell migration [136].

Lastly, DDR1 can associate with TM4SF1 to induce metastatic tumor colonization as described above [26]. No crosstalk between DDR2 and other membrane receptors was found; however, in MCF-7 and NIH-3T3 cells, DDR2 is phosphorylated on tyrosine residues after IGF-2 or insulin stimulation, suggesting possible crosstalk between IGF-1R or/and IR and DDR2 [137].

## 3. Cancer Treatment

DDRs are upregulated in many cancers and appear to be promising biomarkers; thus, their inhibition an attractive strategy. DDR1 and DDR2 can be targeted by tyrosine kinase inhibitors such as dasatinib, nilotinib, and imatinib [139]. These inhibitors have been shown to be effective on multiple occasions. Dasatinib inhibited DDR1 in lung [54] and gastric cancers [140] and DDR2 in studies of head and neck cancers [141]. Similarly, nilotinib inhibited DDR1 in breast and colon cancer studies [60,61]. Although these compounds are effective in inhibiting DDRs, they have broad specificities and, thus, also inhibit other TKRs. Sitravatinib (MGCD-156) is a multitarget TKR inhibitor that can inhibit DDR2 [142].

Table 5 summarizes the imatinib, dasatinib, nilotinib, and sitravatinib specificities against different TKRs and their use in clinical trials targeting DDR1 or/and DDR2.

In recent years, several DDR1-specific inhibitors have been developed. In 2013, the pyrazolo pyrimidin benzamide 7rh and 7rj selective DDR1 inhibitors were discovered. They can inhibit the enzyme activity with IC_50_ (Half-maximal inhibitory concentration) values of 6.8 and 7.0 nM, respectively [57]. In vitro and in vivo studies have shown 7rh effectiveness for pancreatic [143], gastric [72], and lung [144] cancers. KST9046, with a quinazoline urea scaffold, and 8v, a 3′-(imidazo[1,2-a]pyrazin-3-yl)-[1,1′-biphenyl]-3-carboxamide compound, were designed and optimized to inhibit DDR1 [145,146]. DDR1-IN-1 and DDR1-IN-2, two derivatives of the DDR1-interacting structure of imatinib, are capable of inhibiting DDR1 phosphorylation with EC_50_ (Half maximal effective concentration) values of 86 nM and 9 nM, respectively [147]. DDR1-IN-1 inhibits proliferation, migration, and invasion, and it increases apoptosis of prostate cancer cells [101]. Recently, a selective DDR1/DDR2 inhibitor called 2.45 was identified using a parallel DNA encoded library. The 2.45 compound preserves renal function and reduces tissue damage in mice with Alport syndrome, a syndrome in which DDR1 is involved [148]. DDR1 inhibition may be a promising solution in some cancers; however, in pancreatic cancer, long-term inhibition of DDR1 appears to lead to organ atrophy, as DDR1 is crucial for tissue homeostasis [55]. Furthermore, in some cancers such as renal cancer, DDR1 is downregulated in cancerous tissues and, therefore, its inhibition does not seem appropriate in this case [41].

Fewer compounds are known that can specifically and selectively inhibit DDR2. Actinomycin D, the potent mammalian transcription inhibitor, can impair the interaction between DDR2 and collagen, preventing DDR2 activation [149]. Compound 1 can inhibit DDR2 phosphorylation but without affecting NSCLC proliferation [150]. WRG-28 inhibits the interaction between DDR2 and collagen through allosteric modulation. WRG-28 impedes breast cancer cell migration and invasion in vitro and inhibits lung metastasis formation after injection of tumor cells in the mouse tail vein [117]. Other ways to inhibit DDR2 activity include the use of an anti-DDR2 antibody or a recombinant soluble DDR2 form, which prevents collagen binding to DDR2 [79].

Today, the difficulty with patient management lies in the tumor heterogeneity that does not allow a universal treatment, as well as in the lack of effective treatment, which leads to the appearance of resistance and cancer relapse. Some researchers have highlighted the role of DDR1 in treatment resistance in lung [144], breast [151], pancreas [143], glioma [152], ovarian [153], and head and neck cancers [154]. For example, in glioblastoma, DDR1 drives chemoresistance by collaborating with the 14-3-3/BECN1 (Beclin 1)/AKT1 multiprotein complex to enhance cell survival, anti-autophagy, and resistance by AKT/mTOR signaling [152]. However, DDR1 can improve the chemosensitivity of prostate cancer cells, with DDR1b increasing the sensitivity of cells to doxorubicin and paclitaxel [155].

DDR2 is also involved in treatment resistance. DDR2 has been identified as a critical target for improved response to anti-PD1 (Programmed death-ligand 1) immunotherapy. The combination of anti-PD1 therapy and DDR2 inhibition with dasatinib shows a reduction in tumor burden and results in an increase in CD8^+^ T-cell counts in bladder, breast, colon, and sarcoma cancers. Melanoma metastasis in mouse lungs is largely inhibited if mice receive both treatments in combination [156]. Despite the demonstrated efficacy in inhibiting DDR2, acquired resistance to dasatinib can occur through acquisition of the DDR2 gatekeeper mutation T654I in NSCLC [157]. On the other hand, other mutations such as L595P lead to increased sensitivity to dasatinib in squamous cell lung cancer [70]. No specific DDR inhibitors are in clinical trials. Table 6 summarizes the specificities of the different DDR inhibitors and their use in preclinical assays.

## 4. Discussion

DDRs are involved in different stages of tumor development and metastasis. No evidence has been observed for their involvement in tumor initiation, but DDR1 and DDR2 play a role in tumor cell proliferation (Figure 1c) and more particularly in TME remodeling (Figure 2a). Both receptors are important for MMP expression, collagen fiber alignment, and immune cell migration and activation, whilst DDR2 is important for CAF activity. They are also associated with tumor angiogenesis and lymphangiogenesis, both processes enabling tumor cell intravasation (Figure 2b). In addition, DDRs are also important in tumor cell migration and invasion. DDR1 is involved in collective migration and, certainly, in individual migration via EMT induction (Figure 2a). After intravasation, no evidence for the involvement of DDRs in the survival of circulating tumor cells was found (Figure 3a). Nevertheless, some collagens and other noncanonical ligands (e.g., C1q) are found in the serum of cancer patients. Furthermore, DDR1 increases cell–cell adhesion; hence, we can hypothesize a role of DDR1 in the adhesion of tumor cells to protective cells such as platelets, CAFs, or immune cells. No direct evidence for the involvement of DDRs in extravasation has been found, although there are clues for roles of DDR1 and DDR2 in extravasation in bone and liver, respectively. The metastatic niche plays a crucial role in the formation of micrometastases (Figure 3c). The role of DDRs in the metastatic niche depends on the metastatic tissue and on the DDR isoform. DDR1, but not DDR2, is important for the formation of the liver niche, whereas DDR2 is necessary for the formation of the lung niche. Only DDR1, in association with TM4SF1, is essential for the development of micrometastases in macrometastases (Figure 3c).

In almost all stages of metastasis, apparently contradictory results have been obtained. Different hypotheses may explain the contrasted effects of DDR1 and DDR2 manipulation on proliferation, EMT, MMP induction, migration, invasion, and metastasis. First, DDR1 activity may depend on the type of cell and of the presence or absence of an interacting protein (such as DARPP-32) [118]. Second, DDR1 and DDR2 activity may be assay-dependent. DDRs induce cell proliferation in a 2D assay; however, in a 3D assay, DDRs inhibit cell proliferation [60]. Third, the activity of DDR1 activities may be isoform-dependent. As mentioned above, DDR1 has five different isoforms, the most commonly studied being DDR1a and DDR1b, although many studies do not mention or distinguish them. In pancreatic cancer, DDR1b is able to upregulate N-cadherin in a collagen-dependent manner. This regulation is due to the interaction between the phosphotyrosine domain of the phosphatase Shc1 (SHC Adaptor Protein 1) and Tyr513, absent in DDR1a or DDR1b [158]. On the other hand, in glioma, DDR1a is able to induce invasion, adhesion, and MMP2 activation [159] and, in NSCLC, DDR1a has a stronger effect in cell migration and invasion than DDR1b [160]. A switch between DDR1 and DDR2 during tumor progression has been demonstrated, but it is not impossible that a switch between the different DDR1 isoforms during tumor progression may exist. These important aspects remain to be elucidated, and this will be important for a better adaptation of treatments in different cancers.

Most of the late metastasis cascade (i.e., blood survival, extravasation, micrometastasis, and macrometastasis) has not been studied individually but as a whole after injection of tumor cells into the bloodstream. It will be interesting to clarify in which step(s) DDRs are involved.

There are not many studies that focused on the analysis of the impact of DDR1 and DDR2 within the same cancer, although, very often, both receptors are expressed. Further studies analyzing the relationship between the two receptors in tumor development are needed.

DDR inhibitors used in cancer treatment, such as dasatinib or nilotinib, are not specific DDR inhibitors. Further in vivo analysis of cancer development during treatment using specific DDR1 or DDR2 inhibitors, such as 7rh, 2.45, or WRG-28, in different cancers are needed.

## 5. Conclusions

DDRs are receptors for ECM collagens. They are expressed at the extracellular membrane of tumor cells and of many cells of the TME such as CAFs and immune cells. In many cancers, DDR1 or DDR2 are overexpressed, suggesting a role for DDRs in tumor development and metastasis. They are involved in the proliferation and migration of tumor cells and in the formation of micro- and macrometastases. However, the impact of DDRs in tumor development or metastasis seems to be cancer-dependent and may be explained by the DDR1 isoform expressed or by interaction with other receptors. Further experiments are needed to clarify the activities of DDRs in different cancers. These studies will be facilitated by the development of specific DDR inhibitors that are being characterized in vitro and in vivo.

## Figures and Tables

**Table 2:** Roles of DDRs in matrix metalloproteinase (MMP) activation and invadosome formation.

Receptor	MMP Activation/Invadosome Formation	Cancers
DDR1	Increase	-Lung carcinoma [82] -Pituitary adenoma [83] -Colorectal carcinoma [84] -Renal carcinoma [85] -Pancreatic [86] -Liver [87] -Breast carcinoma [24]
	Decrease	
DDR2	Increase	-Ovarian [32] -Melanoma [62]-Hepatocellular carcinoma [89] -Breast [90]
	Decrease	

**Table 3:** Roles of DDRs in the mechanism of EMT.

Receptor	EMT	Cancers
DDR1	Increase	-Prostate [101] -Gastric [102,103] -Hepatocellular carcinoma [35] -Colorectal [84] -Renal carcinoma [85]
	Decrease	-Breast carcinoma [31,104,105] -Ovarian [45,104] -Liver (female, [104])
DDR2	Increase	-Breast [30,90,98] -Ovarian [32] -Papillary thyroid carcinoma [107] -Hepatocellular carcinoma [89] -Gastric [67]
	Decrease	

**Table 4:** Roles of DDRs in tumor cell migration and/or invasion.

Receptor	Migration/Invasion	Cancers
DDR1	Increase	-Squamous cell carcinoma [25,112] -Hepatocellular carcinoma [35] -Pancreatic [86] -Breast [113] -Renal carcinoma [85] -Gastric [103] -Prostate [53] -Colorectal [120] -Epidermoid carcinoma [48]
	Decrease	-Breast [118] -Pancreatic [114]
DDR2	Increase	-Gastric [64] -Hepatocellular carcinoma [65] -Melanoma [115] -Squamous cell carcinoma [63,116] -Breast [99,117] -Ovarian [32]
	Decrease	

**Table 5:** Nonspecific DDR inhibitors: specificities and clinical trials (adapted from ClinicalTrials.gov).

Inhibitor	Targeted Kinases	In Vitro Assay (Cancer)	Preclinical (Cancer)	Clinical (DDR Targeted)
Imatinib	BCR-ABL, DDR1, DDR2			
Nilotinib	ABL1, Kit, PDGFRA, PDGFRB, CSF1R, DDR1, DDR2	DDR1: -Breast (decreased apoptosis and DDR1 phosphorylation) -Colon (increased proliferation, decreased DDR1 phosphorylation).		-NCT02029001: Malignant solid neoplasms. Mutated DDR1, mutated DDR2. Phase 2 (recruiting).
Dasatinib	BCR-ABL, SRC kinase family, DDR1, DDR2	DDR1: -Lung (decreased proliferation). -Gastric (decreased proliferation, migration, invasion). DDR2: -Head and neck (decreased proliferation, migration, invasion)	DDR2: -Head and neck (zebrafish): decreased migration	-NCT01491633: Squamous cell lung cancer. Mutated DDR2. Phase 2 (not evaluable, toxicity). -NCT04439305: lymphoma, myeloma, solid neoplasm. Mutated DDR2. Phase 2: withdrawn. -NCT01514864: NSCLC. Mutated DDR2. Phase 2: not completed, disease progression.
Sitravatinib (MGCD156)	MET, AXL, VEGFR1, VEGFR2, VEGFR3, PDGFRA, PDGFRB, TRKA, TRKB, DDR2			-NCT02219711: NSCLC, renal cell carcinoma. Mutated DDR2. Phase 1 (recruiting).

**Table 6:** Specific DDR inhibitors and some examples of their used in preclinical assays. Specificity describes the strength of binding between a receptor and an inhibitor and it is measured by the dissociation constant (*K_D_*).

Inhibitor	Specificity	In Vitro Assay (Cancer)	Preclinical (Cancer)
7rh	DDR1 > DDR2	-Pancreatic (decreased DDR1 phosphorylation, signaling, colony formation, migration) -Gastric (decreased proliferation) -Lung (increased apoptosis)	-Pancreatic (mice): decreased tumor development and proliferation, increased apoptosis and survival (in association with chemotherapy). -Gastric (mice): decreased peritoneal tumor nodules. -Lung (mice): In association with chemotherapy, decreased tumor development and increased apoptosis
7rj	DDR1 > DDR2		
KST9046	DDR1 > ARAF, FLT3, LIMK1, LMK2	-Decreased proliferation (leukemia, lung, colon, brain, melanoma, renal, ovarian, prostate and breast) and DDR1 phosphorylation	
8v	DDR1 > DDR2, BCL-ABL, c-KIT	-Lung: decreased colony formation, proliferation, migration, invasion and DDR1 phosphorylation	
DDR1-IN1	DDR1 > DDR2	-Decreased DDR1 phosphorylation, cell proliferation (colon, breast, lung, uterus, liver). -Prostate: decreased proliferation, cell viability, colony formation, EMT and migration. Increased apoptosis.	
DDR1-IN2	DDR1 > DDR2	Decreased DDR1 phosphorylation, cell proliferation (colon, breast, lung, uterus, liver)	
2.45	DDR1 > DDR2	Decreased DDR1 phosphorylation	
Compound 1	DDR2	Decreased DDR2 phosphorylation and lung cell proliferation.	
WRG-28	DDR2	Breast: decreased migration and invasion.	Decreased breast metastasis to lung

## Data Availability

No new data were created or analyzed in this study. Data sharing is not applicable to this article.
